# Molecular diversity of Protura in southern High Appalachian leaf litter

**DOI:** 10.3897/BDJ.11.e113342

**Published:** 2023-11-24

**Authors:** Michael Caterino, Ernesto Recuero

**Affiliations:** 1 Clemson University, Clemson, United States of America Clemson University Clemson United States of America

**Keywords:** Protura, soil biodiversity, megabarcoding, Appalachia, species delimitation

## Abstract

The higher elevations of the southern Appalachian Mountains, U.S.A., host a rich, but little-studied fauna of Proturan hexapods. Here, we publish 117 Proturan barcode sequences from this region, estimated by automated species delimitation methods to represent 72 distinct species, whereas only nine species have previously been reported from the region. Two families, Eosentomidae and Acerentomidae, co-occur at most sampling sites, with as many as five species occurring in sympatry. Most populations exhibit very low haplotype diversity, but divergences amongst populations and amongst closely-related species are very high, a finding common to other phylogeographic studies of Proturans. Though we were unable to identify any of the barcodes to species, they form a useful, if preliminary, glimpse of southern Appalachian Proturan diversity.

## Introduction

The Protura, or 'coneheads', are an order of minute litter and soil-inhabiting hexapods. They are poorly known and little studied, but are generally considered to be general detritivores, though a number of species have been observed to feed more directly in mycorrhizal fungi (Sturm 1959, Pass and Szucsich 2011, Bluhm et al. 2019). The group has a relatively low diversity, with around 800 species described from all continents, but Antarctica (Galli and Rellini 2020). The North American fauna of this group was reviewed by Allen (2007), who provided an annotated catalogue and some identification keys (species of *Eosentomon* Berlese 1908), building on an earlier regional synthesis by Ewing (1940). There are very few active specialists in the group, not only in North America (e.g. EC Bernard), but worldwide (Bernard and Whittington 2021) and much taxonomic and ecological work to be done on them.

The Protura fauna of the southeastern US has not received any specific attention. Allen's (Allen 2007) checklist indicates that 12 genera and 39 species, from three different families (Eosentomidae Berlese, 1909, Acerentomidae Silvestri, 1907 and Protentomidae Mills, 1932) are known from the southeastern region generally, most from only their type localities.

In this paper, we report the results for Protura of a molecular-barcoding project focused on the arthropods occurring in leaf litter of the higher elevations of the southern Appalachian Mountains. Most elevations above 1500 m in this region host a distinctive coniferous forest type dominated by red spruce (*Picearubens* Sarg.) and Fraser fir (*Abiesfraseri* [Pursh.] Poiret). The arthropods of these sky islands have been little documented and the Protura not at all. Of the 39 species reported from somewhere in the southeastern USA, nine have been more or less explicitly reported from the southern Appalachians Mountains (Allen 2007). None of these is represented by COI barcode sequences in any public databases. Although none of the sequences we report here is identified beyond the family level, they provide a unique snapshot of the region's genetic diversity of Proturans and offer a novel perspective on ranges and distributions of monophyletic lineages in the area.

## Material and methods

**Figure 1.**
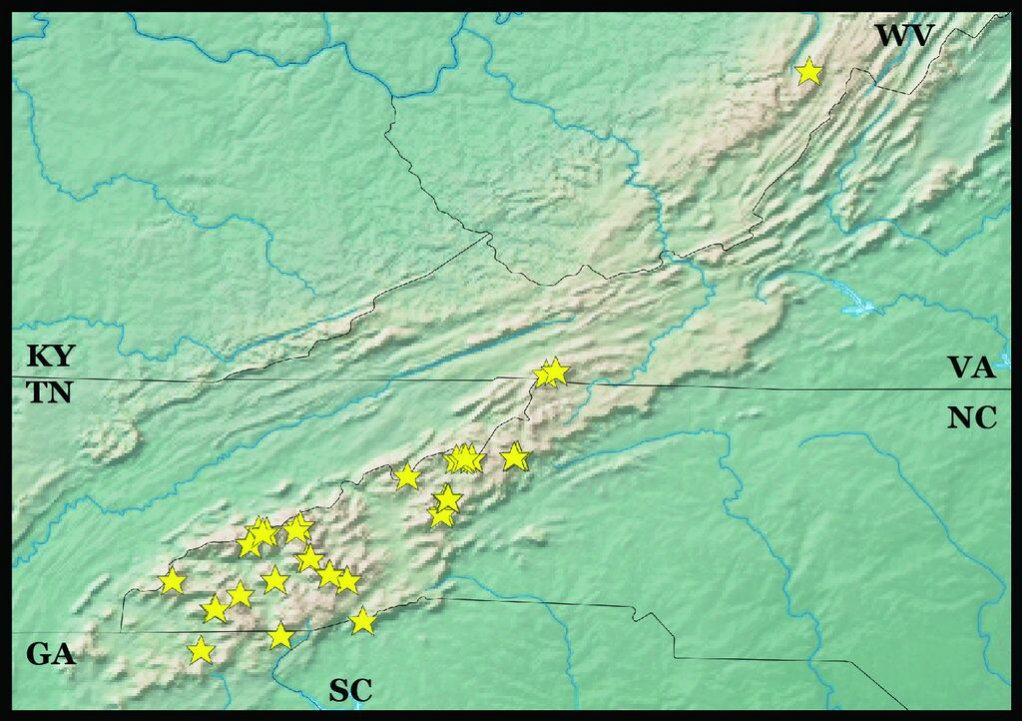
Localities of barcoded Proturan sequences in the southern Appalachian Mountains.

Samples were collected across southern Appalachia using an 8 mm mesh litter sifter with subsequent processing in Berlese funnels. Litters targeted were predominantly those of the high elevation coniferous spruce-fir forest floor, but lower elevation samples were more typically broadleaf deciduous and evergreen (*Rhododendron* L.) litters. See Fig. 1 for sampling sites and Suppl. material 2 for all sampling site data. Arthropods were collected directly into 100% ethanol and specimens sorted to unique morphotypes to which we refer as morphospecies. Prior to extraction, each specimen was photographed (https://www.flickr.com/photos/183480085@N02/albums/72157710333048247), then punctured. Following digestion with proteinase K, vouchers were recovered where possible (stored in 95% ethanol with a few drops of 100% propylene glycol) and the liquid fraction was purified using magnetic bead extraction. A COI minibarcode was amplified using primers BF2-BR2 (Elbrecht and Leese 2017), tagged with a unique combination of 9 bp indexes (see Meier et al. (2015)) and sequenced on either Illumina or Nanopore platforms, purifying and adding adapters following protocols specific to each. Sequences were demultiplexed and quality-checked to obtain a single barcode for each specimen. For more complete details of laboratory and analytical procedures, please see Caterino and Recuero (2023). Recovered vouchers are housed in the Clemson University Arthropod Collection. Appendix 2 includes full label and voucher code data, as well as GenBank accession numbers.

To summarise similarities amongst barcoded specimens, we conducted Neighbour-joining analyses in PAUP*, using Kimura 2-parameter distances (Kimura 1980). Preliminary hypotheses of species richness were inferred using Assemble Species by Automatic Partitioning (ASAP; Puillandre et al. (2020)), also with Kimura 2-parameter distance correction and mPTP (Kapli et al. 2017), using a tree generated with BEAST 1.10.4 (Suchard et al. 2018), using a strict molecular clock and a Yule tree prior, running for 10 million generations, sampling every 1000. As a secondary attempt to place Appalachian taxa in a broader taxonomic context, we downloaded available barcoding region sequences from GenBank and aligned those with ours, producing a matrix of 198 Proturan sequences. Relationships amongst the sampled populations were analysed with W-IQ-Tree (Trifinopoulos et al. 2016) under Maximum Likelihood criteria, using its automated model selection to choose GTR+gamma, with empirical base frequencies. All trees were rooted with a Dipluran (Campodeidae Meinert 1865) outgroup.

## Data resources

The data resources associated with this paper comprise a nexus file including all new barcode sequences (Suppl. material 1) and an Excel file (Suppl. material 2) with all associated voucher data, unique identifiers and GenBank codes.

## Results

We extracted 136 Proturan specimens from our samples for barcoding, 117 of which yielded barcode sequences (success rate 86%). Eleven voucher specimens were recovered following extraction.

**Figure 2.**
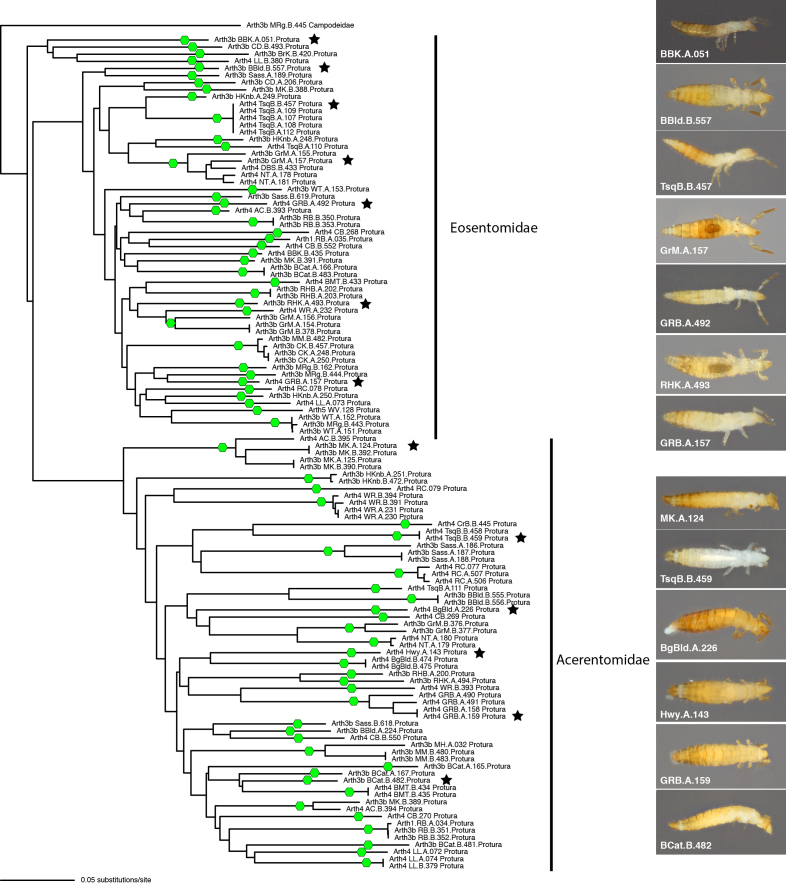
Neighbour-joining tree of Appalachian region barcoding sequences, based on K2P distances. Green hexagons on branches indicate ASAP-inferred species level lineages. Black stars indicate specimens pictured at right.

A Neighbour-joining phylogram, showing degree of similarities amongst sequences, is shown in Fig. 2. They resolve into three main clusters, two of which apparently represent Eosentomidae and one Acerentomidae (based on higher level comparisons not shown, but discussed further below).

**Figure 3.**
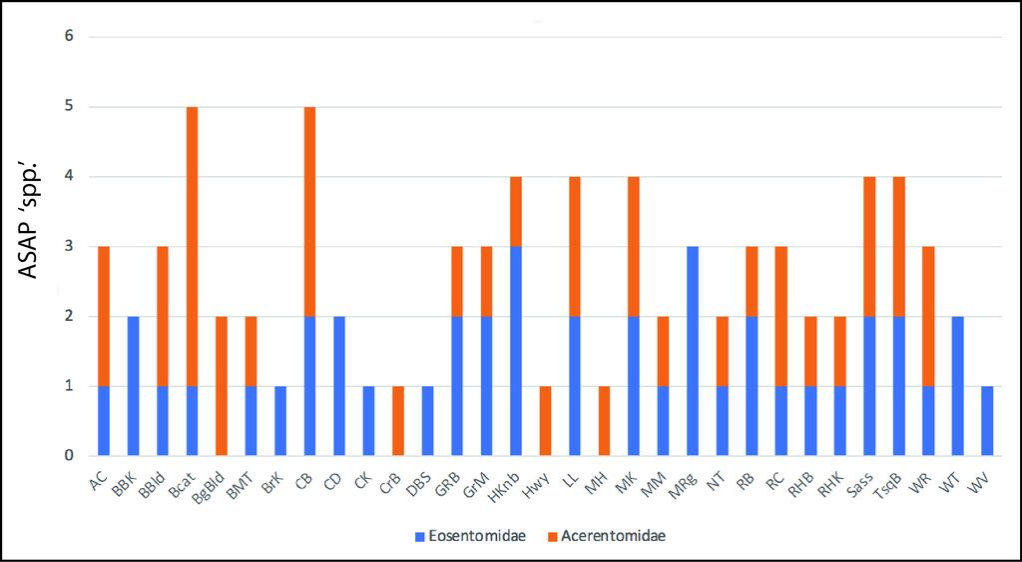
Number of ASAP-estimated species by sampling site in each represented family.

ASAP analyses suggests these 117 sequences to correspond to 72 distinct species, 38 in the Eosentomidae and 34 in the Acerentomidae and a threshold distance of 9.9%. Most studied localities with presence of Protura harbour two of these units (n = 10), but we have found localities with one (n = 7), three (n = 8), four (n = 4) and up to five (n = 2) approximately species-level units (Fig. 3). According to our data, most delimited units have a limited range and are exclusive to a single population and only five of them, two Eosentomidae and three Acerentomidae, are present in more than one sampled locality (three localities at most). The results of mPTP analyses, with 49 delimited species, are more conservative (Suppl. material 3); despite having high genetic distances, divergent haplotypes are often lumped into single hypothetical species. Considering mPTP species, we observe a few widely distributed species, some of then occurring across much of the southern Appalachian Mountains. A more detailed study of morphology might help distinguish between those hypotheses. In the absence of other evidence, these results must be considered as mere approximations; however, given the high distance threshold considered for ASAP results, it is likely that the number of species is closer to our higher estimates.

## Discussion

Diversity of high Appalachian Protura appears to be high, with more than 70 inferred species in two different families over a relatively limited spatial sampling. Nearly all of these putative species are restricted to single sampling sites, with a few exceptions discussed further below. Sequences hypothesised to be conspecific were frequently identical, even from different local samples and dates. Divergences from nearest neighbours, however, were invariably high, 8-10% uncorrected distance or higher. Clusters generally reflect geography, with nearest neighbours very often from nearby peaks or ranges (e.g. CD.A.206 and MK.B.388, from Clingmans Dome and Mt. Kephart, separated by ~ 4 km on either side of the Newfound Gap in the Smokies). Similar patterns of very low intrapopulational variation in combination with high interpopulation and interspecific distances have been reported in other species-level studies of the group (Resch et al. 2014), suggesting some interesting population level dynamics.

Nearly all localities are furthermore represented by multiple distinct species and lineages, indicating a complex fauna with a long history of mixing. Big Cataloochee and Cowee Bald samples contain five species, while Huckleberry Knob, Tusquitee Bald, Sassafras Mt. and Mt. Kephart each have four. Thus, local faunas of Protura may be reasonably rich. We must also acknowledge the likelihood that most of our sites are likely still undersampled; particularly, by selecting only a single specimen per morphospecies per population, we may have overlooked morphologically similar or even cryptic species that may co-exist in the same locality.

Only a few species range beyond single sites. One putative species occurs not only on one of Grandfather Mountain's summits (Calloway Peak), but also on its lower slopes (Daniel Boone Scout and Nuwati Trails – though in another lineage that spans these sites, these form a more divergent sister species pair). A species in the Black Mountains was collected both on the summit of Mount Mitchell and on Celo Knob, 10 km apart. Another Black Mountains species was found on both Mount Mitchell and Mt. Hallback (2 km apart). A species was shared by Mt. Rogers and Whitetop, in southwestern Virginia, spanning 6 km. To some degree, these results will have been impacted by the density of sampling and it is conceivable that more species sharing across localities would be suggested if intervening localities were better sampled. However, it should not be surprising for such minute, relatively immobile and environmentally sensitive animals to be highly isolated and, consequently, highly diverse in such varied topography and environments.

There are limited clear biogeographic signals in these sequences and it is likely that many of these broader distributions predate many of the environmental fluctuations of the Pleistocene. Any larger cluster includes representatives from both sides of the Asheville Depression, typically observed as a major biogeographic breakpoint in the region (e.g.Browne and Ferree 2007, Hedin et al. 2015, Caterino and Langton-Myers 2019, Caterino in press). However, resolution that might reveal more significant biogeographic relationships is also limited by deep divergences and saturated signal in such a short mitochondrial fragment. More extensive sequencing of a similar set of taxa might be expected to show more consistent geographic signal.

Our sequences apparently represent the families Eosentomidae and Acerentomidae, as indicated in Fig. 2. This is based on nesting within clades of identified sequences of both, predominantly Asian species (reported in Bu and Wu 2012, Bu and Bai 2013, Shrubovych et al. 2014a, Bu et al. 2017, Qian et al. 2018, Carapelli et al. 2019) and a few Neotropical Acerentomidae (*Andinentulus* Tuxen, 1984; Shrubovych et al. (2014b)). Though most are quite distant from southern Appalachian sequences, one of seven (unidentified) Eosentomidae sequences from eastern Canada available in the BOLD database resolves within our Appalachian species, as sister to a sequence from Whitetop Mt. in Virginia and one Asian *Eosentomonnivoculum* Yin 1981 sequence is resolved as sister to a rather divergent clade of sequences from the Great Smoky, Plott Balsam and Great Balsam Mts. (including CD.B.493, BBK.A.051, BrK.B.420 and LL.B.380).

This study represents the first view, limited though it is, of Proturan diversity in the southern Appalachian Mountains. As in many groups, this biodiversity hotspot appears to host a wealth of Proturan species, at least twice as much (and likely several times more) species richness than is yet reported. One challenge with a barcode-based approach, such as we report here, will be corresponding morphological work. Only a small number of voucher specimens (11) were recoverable following extraction. Between their small size and near transparency after they are digested with proteinase K, it is very difficult to avoid pipetting or otherwise overlooking the specimens themselves. It is also apparent that a morphospecies-based presorting is a very poor approximation to species. Where we compare pre-extraction photographs of putative DNA-based conspecifics, we see little consistency, with sorting frequently misled by varied degrees of sclerotisation, distension and other taxonomically meaningless artefacts. Bridging the gap to forge an integrative taxonomy of Proturans will require much more careful approaches (such as detailed in Böhm et al. (2011), Resch et al. (2014)). However, as a sorely neglected and likely megadiverse lineage, the results should repay the efforts.

## Supplementary Material

555D642F-C4C7-55E9-9657-F35C1E5E6DCD 10.3897/BDJ.11.e113342.suppl1 Supplementary material 1 Cytochrome Oxidase I barcode region sequences for Appalachian Protura Data type phylogenetic Brief description A total of 117 partial COI sequences of Protura from the southern Appalachians, in nexus format. File: oo_912190.nex https://binary.pensoft.net/file/912190 Caterino MS, Recuero E

7AA96B70-79FA-5649-BCAF-BA4CB1D32B31 10.3897/BDJ.11.e113342.suppl2 Supplementary material 2 Collecting and voucher information for Proturan barcode sequences Data type occurrence Brief description An Excel spreadsheet containing specimen collecting data (locality, date, lat/lon), voucher codes, DNA extraction codes and GenBank accession numbers for all sequences reported. File: oo_940470.xlsx https://binary.pensoft.net/file/940470 Caterino MS, Recuero E

7486556B-5555-544E-8883-E648A98ABFF9 10.3897/BDJ.11.e113342.suppl3 Supplementary material 3 Species delimitation by mPTP. Tree generated by BEAST. Data type phylogenetic hypothesis Brief description Bayesian topology generated by BEAST for mPTP species delimitation analysis. Clades in red represent collections of specimens united as distinct putative species hypotheses, while terminal branches in green indicate putative species represented by single OTUs. File: oo_928958.pdf https://binary.pensoft.net/file/928958 Caterino MS, Recuero E
