# Supplementary figures and images for: Molecular diversity of Protura in southern High Appalachian leaf litter
Source: Biodivers Data J. 2023 Nov 24;11:e113342. doi: 10.3897/BDJ.11.e113342 (PMC10838044; doi:10.3897/BDJ.11.e113342)

0.1268662

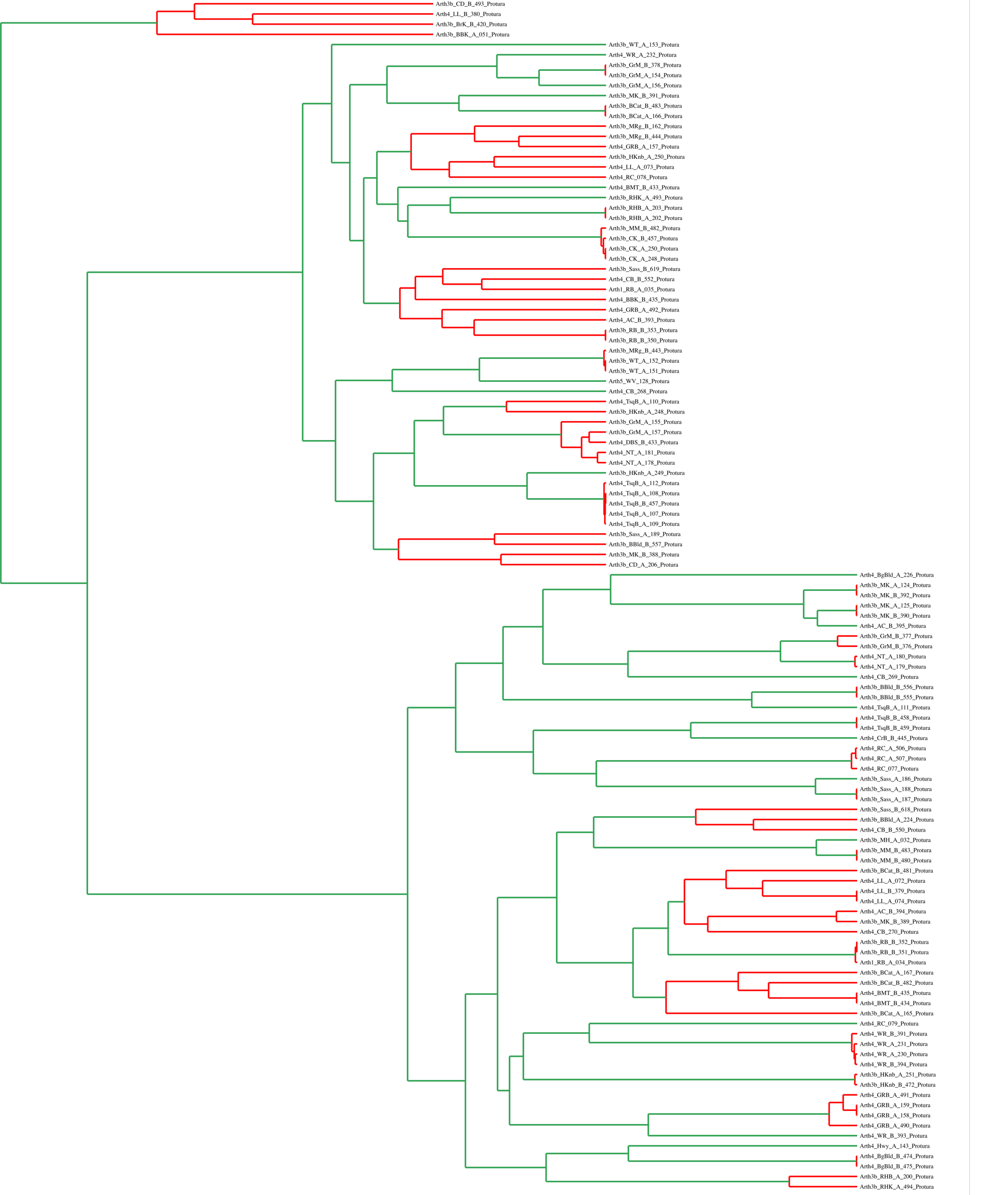

Supplement: Supplementary material 3 — Species delimitation by mPTP. Tree generated by BEAST. [file bdj-11-e113342-s003.pdf]
